# An Interdisciplinary Overview on Ambient Assisted Living Systems for Health Monitoring at Home: Trade-Offs and Challenges

**DOI:** 10.3390/s25030853

**Published:** 2025-01-30

**Authors:** Baraa Zieni, Matthew A. Ritchie, Anna Maria Mandalari, Francesca Boem

**Affiliations:** Department of Electronic and Electrical Engineering, University College London, London WC1E 7JE, UK; a.mandalari@ucl.ac.uk (A.M.M.); f.boem@ucl.ac.uk (F.B.)

**Keywords:** sensing, IoT, AAL, healthcare, privacy, decision-making system

## Abstract

The integration of IoT and Ambient Assisted Living (AAL) enables discreet real-time health monitoring in home environments, offering significant potential for personalized and preventative care. However, challenges persist in balancing privacy, cost, usability, and system reliability. This paper provides an overview of recent advancements in sensor and IoT technologies for assisted living, with a focus on elderly individuals living independently. It categorizes sensor types and technologies that enhance healthcare delivery and explores an interdisciplinary framework encompassing sensing, communication, and decision-making systems. Through this analysis, this paper highlights current applications, identifies emerging challenges, and pinpoints critical areas for future research. This paper aims to inform ongoing discourse and advocate for interdisciplinary approaches in system design to address existing trade-offs and optimize performance.

## 1. Introduction

The Internet of Things (IoT) and AAL technologies have revolutionized healthcare by facilitating continuous health monitoring and emergency detection through various sensors. Wearable devices, such as smart watches and fitness trackers, track activity patterns to identify health issues, particularly for vulnerable populations like the elderly and stroke survivors, while ambient acoustic sensing enhances remote patient monitoring [1,2,3,4,5]. A key application of AAL systems in elderly care is activity recognition and location tracking [6,7]. These systems also support individuals with cognitive impairments, fostering autonomy and independence in daily activities [8]. Additionally, AAL devices like biosensors enable continuous monitoring of vital signs, including heart rate, blood pressure, and oxygen saturation, providing essential health insights, while smart pill dispensers enhance medication adherence for elderly and chronically ill individuals [9,10,11,12,13]. The increasing use of biosensors, particularly in wearable devices, has offered cost-effective and sensitive solutions for detecting physiological parameters like stress and fatigue [10,11,14]. Moreover, the shift toward home-centric healthcare highlights the adoption of unobtrusive sensors, which allow for continuous monitoring without requiring user interaction, providing a distinct advantage over wearable devices that need regular maintenance, such as recharging [15,16]. This aligns with the broader trend in healthcare toward proactive, predictive approaches, where biosensors help to establish biological norms and detect deviations, enabling more precise and personalized treatments [17].

AAL systems hold immense potential for improving home-based chronic disease management, rehabilitation, and elderly care by enabling continuous monitoring and timely interventions. Wearable sensors and radar-based systems have been employed in clinical trials for gait analysis and fall detection, enabling the real-time monitoring of elderly individuals. Studies have shown that such systems are particularly effective in identifying symptoms of sarcopenia, such as slow gait and poor balance, which are linked to an increased risk of falls and impaired physical performance [18,19]. Discreet real-time health monitoring through IoT sensors has the potential to transform personalized and preventative care, particularly in private settings like homes [20]. However, deploying such systems raises complex challenges around data management, privacy, security, cost, and overall reliability. The field of health monitoring has evolved significantly, with distinct research communities addressing various aspects under the broad macroareas of health engineering, sensing, communication, control, and signal processing. Each of these macroareas focuses on specific problems, methodologies, and technologies. For instance, prior studies have explored individual aspects of at-home sensing methods [21,22,23], IoT privacy concerns [24], and decision making within connected systems [25,26], and there is a notable gap in a unified framework that integrates these aspects effectively for real-world application.

Most of the general reviews on AAL systems were conducted prior to 2021, with more recent literature focusing on specific aspects or diseases addressed by these systems. Early reviews offered a broad overview of the AAL ecosystem, emphasizing the importance of addressing stakeholder needs rather than simply adopting emerging technologies [27]. Several reviews examined the development of AAL systems designed for elderly populations and computational techniques, highlighting their potential to promote independent living [28,29,30,31,32]. More recent studies have shifted their focus to IoT-supported AAL systems [33,34], explored the connections between technological advancements and related healthcare needs [35], and identified limitations in the application of machine learning within AAL systems [36].

Despite the breadth of these reviews, none comprehensively address the challenges of AAL systems from integrated perspectives of sensing, communication, and decision making. Such a holistic, systems-level approach is vital for designing robust AAL systems capable of addressing the trade-offs and interdependencies inherent in this interdisciplinary domain. Previous reviews have predominantly examined these aspects in isolation, failing to provide a unified view of how these core disciplines interact to influence the design and functionality of AAL systems.

Designing effective AAL systems requires a coordinated approach to integrating sensing, communication, and decision-making components. While each of these components contributes to system performance, their integration presents new challenges, particularly in balancing trade-offs that affect critical attributes such as privacy, response time, and energy efficiency. Effective system design and operation demand collaboration across these domains to navigate these interdependencies. Solutions often target isolated issues, such as enhancing privacy through encryption or reducing sensor power consumption. However, these strategies can introduce trade-offs that undermine overall system performance. For instance, excessive encryption for privacy may delay critical health alerts, and aggressive energy-saving measures may compromise the timely detection of emergencies, like falls or sudden health deteriorations.

The innovation of our work lies in addressing the interconnected and complex and fuzzy challenges in the AAL field through a holistic and interdisciplinary approach. Specifically, we focus on key challenges such as privacy, performance, and usability, and examine them across three critical dimensions: sensors, communication systems, and decision-making processes. Unlike a traditional literature review, our framework goes beyond summarizing existing research by integrating these dimensions into a unified perspective that highlights their interdependencies and trade-offs. This approach provides actionable insights for designing efficient, secure, and user-centered AAL systems, offering a roadmap for navigating the inherent complexities of the field.

This paper aims to clarify these complex inter-related challenges by presenting an interdisciplinary perspective on the design and operation of AAL systems. Section 2 details the challenges and classifications of AAL sensors, Section 3 covers communication and data management considerations, and Section 4 explores the AAL decision-making processes. The interdisciplinary approach discussed in Section 5 underscores the necessity of balancing these trade-offs to advance the field.

This work is guided by two primary research objectives. First, we aim to identify and evaluate recent advancements in the field of AAL and their current applications (RQ1). Second, we seek to examine the interdisciplinary challenges and trade-offs that influence the innovation and deployment of these technologies (RQ2). This paper does not aim for an exhaustive analysis within any one discipline but instead provides a broad perspective to highlight areas in need of further investigation, encouraging future research across various disciplines to address these challenges. The inclusion and exclusion criteria for our review were based on the selection of peer-reviewed and highly cited articles across three core disciplines: sensing in AAL, communication in AAL systems, and decision making in AAL. Priority was given to works that address interdisciplinary challenges and trade-offs rather than those focusing solely on advancements within isolated domains. To achieve this, we first reviewed the abstracts and conclusions of the highly cited papers to assess their relevance and alignment with the review’s objectives. Additionally, we included overview papers that highlight challenges and complexities within these interconnected fields.

To identify broader surveys and reviews relevant to AAL systems, we conducted targeted searches using terms such as “Ambient Assisted Living healthcare survey/review” in Google Scholar. While our approach did not rely exclusively on keyword-based searches, we prioritized the literature that provides holistic insights into the interplay between sensing, communication, and decision making in AAL.

The gathered literature was carefully analyzed to identify recurring themes, insights, and gaps related to the integration of these components. This synthesis informed the overall insights presented in the manuscript, particularly regarding how trade-offs and interdependencies impact system performance. By emphasizing these aspects, our review offers a comprehensive perspective that addresses the complexities of developing effective, robust, and privacy-enhanced AAL systems.

## 2. AAL Sensors

This section outlines the challenges and classifications of AAL sensors, offering an overview of the data types that they collect. It highlights their primary limitations and discusses the trade-offs inherent in their deployment and utilization.

### 2.1. AAL Sensor Classification

Sensors are categorized into two main types: active and passive. Active sensors, such as LiDAR and radar, emit signals and require an external power source to operate [37]. Passive sensors, such as thermal and electric field sensors, rely on existing environmental signals [38].

For the physical variables considered in the context of AAL and health monitoring, various sensor types may be available to measure the same quantities, each offering different levels of accuracy, sensitivity, and adaptability to different environmental conditions. These differences influence design decisions when optimizing sensor deployment in home settings. Deploying multiple sensors for the same measurements can introduce redundancy, enhancing system robustness and allowing for flexible sensor placement, which can improve the reliability and accuracy of data collection [39,40]. However, redundancy also poses certain trade-offs. The need to process data from multiple sensors can slow real-time decision making, potentially delaying critical interventions. Additionally, excessive redundancy can lead to energy inefficiency, as operating numerous sensors simultaneously consumes more power and reduces overall system responsiveness, particularly in emergency situations [41,42]. Furthermore, incorrect sensor placement may result in data inaccuracies, false alarms, or failure to detect critical health events [43]. To address this, ongoing research is focusing on strategies that optimize sensor placement. Automated calibration techniques are being developed to enhance system performance and reliability by dynamically adjusting sensor positioning [44].

Sensors collect data through three primary methods: contact direct, non-contact direct, and indirect sensing. Each has unique applications, advantages, and challenges, as described below.

#### 2.1.1. Contact Direct Devices (Body-Worn Sensors)

Body-worn sensors (BWSs) monitor health through direct skin contact, using devices like accelerometers, gyroscopes, and magnetic sensors [45]. While effective, their reliance on skin contact can cause discomfort and user compliance issues. Proper placement is critical to avoid motion artifacts and ensure data accuracy, especially for devices like ECGs, EMGs, and EEGs, which require well-designed electrodes to maintain signal quality [46]. However, compliance can be an issue, as users must remember to re-attach sensors after activities like sleeping or showering. Additionally, over 80% of doctors now use mobile devices for patient care, further expanding the touchpoints of healthcare monitoring beyond traditional devices [17,47]. Thus, a sensor systems approach that integrates design, attachment, and data quality is essential for ensuring effective long-term monitoring and user compliance.

#### 2.1.2. Non-Contact Direct Devices (Ambient Sensors)

Non-contact sensors, commonly used in ambient applications, allow for continuous 24-hour monitoring of individuals without requiring direct physical contact. These sensors are widely integrated into home environments to passively observe behavior and activity patterns or to activate devices like lights, often triggered by events such as bed exits during the night. Technologies including active and passive radar, infrared (IR), and camera-based systems are frequently employed for such monitoring. However, camera sensors required ambient light and the subject’s presence within the field of view, raising privacy concerns, especially in sensitive areas [48]. Passive infrared (PIR) devices, one of the most popular non-contact motion sensors, detect infrared radiation and offer a cost-effective, mass-produced solution, though their data fidelity is limited [37]. Other sensors, such as acoustic receivers, pressure pads, and thermal infrared devices, face challenges from environmental interferences like noise or heat sources, and retrofitting them into home settings can be prohibitively expensive [48]. Passive radar sensors show promise for healthcare monitoring, particularly for privacy and security-sensitive applications. However, ensuring their robustness and accuracy in real-life conditions remains challenging as most solutions have only been tested as proof-of-concept devices in controlled environments [49].

#### 2.1.3. In-Direct Devices

Indirect sensors, such as smart meters and pressure and vibration sensors, are essential for non-invasive, continuous monitoring, as they capture critical data without requiring direct physical contact. These sensors are particularly relevant in load monitoring systems, where two primary approaches, intrusive load monitoring (ILM) and non-intrusive load monitoring (NILM), are commonly used. ILM involves multiple sensing points, offering higher accuracy and reliability, while NILM, which uses a single sensing point, simplifies installation and reduces hardware demands but often provides less accurate results, especially in distinguishing energy loads. NILM’s performance is hindered by noisy aggregated signals and limited appliance detection due to sampling frequency constraints, despite advances in artificial intelligence (AI) algorithms. It reliably monitors only major appliances like ovens, washing machines, and HVAC systems, but its effectiveness across different datasets remains inconclusive [50,51,52]. ILM, though more accurate, requires additional equipment, leading to higher costs [53]. Challenges such as long-term reliability, data quality, and processing persist for both approaches.

### 2.2. Data Types in AAL Environment

Data types collected by AAL devices are highly sensitive, covering personal health information, environmental data, and behavioral insights. Understanding the nature of these data types is essential for informed decision making when selecting relevant AAL devices and determining the most suitable data management and communication strategies. IoT data may be classified based on characteristics such as spatial, temporal, and sensory attributes [54], or by its intended usage. While behavioral and functional data can often be monitored straightforwardly, capturing physiological parameters poses additional challenges for unobtrusive measurement within home environments.

**Behavioral Data**: Presence, Activity Duration, and Levels: monitored using ambient sensors such as motion detectors, contact sensors, and pressure sensors.
**Functional Data**: Gait Velocity and Step Time: these are assessed through depth video analysis. Walking Speed: estimated using passive Iifrared motion sensors.
**Bio (Physiological) Data**: Heart Rate: monitored using ballistocardiography (BCG) sensors like pressure or bed sensors, or through electrocardiogram (ECG) electrodes, which can be dry or capacitive. Respiration Rate: tracked via bed sensors. Body Temperature: measured with an infrared thermometer.
**Environmental Data**: Air Quality: Includes gas concentrations and humidity levels, monitored through air sensors. Sound Levels: Captured by sound sensors to assess the ambient noise environment.

### 2.3. Sensing Challenges and Trade-Offs

**Figure 1:**
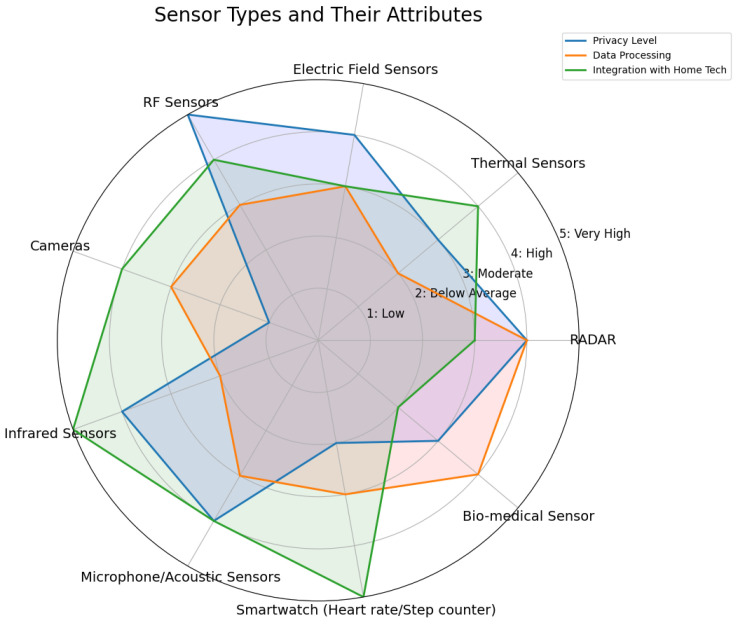
Sensors and privacy.

Privacy remains one of the most pressing challenges in deploying sensing systems within the AAL domain, particularly given the need to balance trade-offs with other system qualities. However, most health sensors lack privacy-centered design considerations [55,56,57]. Privacy concerns in healthcare sensors differ between direct and indirect sensing methods. Direct sensing, such as heart rate monitors, gathers specific data related to a singular measurement, which inherently limits privacy risks by not collecting extraneous information. Conversely, indirect sensing gathers data that can infer the desired information through secondary measurements, often requiring more complex data processing and potentially involving the collection of a broader set of data. Indirect methods like environmental sensors or cameras often collect broader data to infer specific health conditions, inadvertently increasing the risk of exposing sensitive information. Contact sensors like electrocardiograms (ECGs) and galvanic skin response (GSR) devices face additional challenges in terms of user compliance and hygiene, with ECGs benefiting from rapid response times due to electrical signal transmission, while GSRs rely on slower physiological processes [58]. These variations in data processing complexity underscore the need for careful management of both functionality and privacy when utilizing sensors for data capture, particularly when integrating them within AAL technology; see Figure 1. Figure 1 illustrates the relationship between privacy levels, data processing, and integration with home technologies for the primary sensors used in AAL systems. Acoustic sensors, for example, enhance privacy by analyzing auditory patterns, such as falls or unusual sounds, without capturing identifiable personal features. These sensors are designed to detect specific auditory events without retaining raw audio. They effectively capture the necessary data with minimal processing and are seamlessly integrated into other home technologies. In AAL environments, acoustic sensors are widely used to detect events like breaking glass or voice commands, enabling automated responses such as locking doors or sending alerts.

At the device level, the primary challenges revolve around privacy, data integrity [59,60], and secure data handling. Ensuring data usability and integrity while minimizing power consumption remains critical, particularly because frequent encryption and hashing processes can significantly increase energy consumption and computational load. Refs. [61,62]. There is a need for data minimization strategies and anonymisation techniques at the source to avoid the unnecessary collection or storage of personal data without legitimate reasons [63]. Collecting excessive data, like continuous video feeds, can lead to privacy harms. AAL devices should anonymise data before transmission. However, achieving this may directly affect data accuracy and performance [64]. For instance, moving location data from a fitness app can remove critical data of the context needed for health analysis [65]. Overall, integrating privacy measurements throughout the data processing and lifecycle is essential. There is an inherent complexity when trying to address privacy across these system layers, such as balancing the utility of aggregated data, where there is a risk of re-identification or inference of sensitive information with privacy concerns, which is the main challenge.

## 3. Data Management, Storage, and Communication

This section examines the communication protocols commonly used in this domain and explores how these data are managed across homogeneous and heterogeneous network architectures. Additionally, it highlights key challenges within these configurations, including trade-offs in data quality due to varying sensor types and the privacy implications associated with these systems.

### 3.1. Data Management, Network Structure, and Communication

Healthcare monitoring systems employ different data management strategies and network architectures, including homogeneous and heterogeneous sensor networks. Homogeneous networks consist of identical sensors used across a region or system, while heterogeneous networks combine different types of sensors for unified monitoring purposes [66]. Wireless sensor networks (WSNs), which comprise a base station and battery-powered sensor nodes, are a common subset. In WSNs, optimizing energy consumption in sensing, processing, and communication is crucial for extending their lifespan, often through low-power radios and efficient network protocols [17]. These networks can be further classified by application domains, such as health or environmental monitoring, rather than by specific network topologies, like star or point-to-point configurations.

In AAL systems, there are three primary approaches for data storage and processing within sensor networks: (a) cloud-based [67], (b) hybrid [68], and (c) local/edge-based methods [69,70]. Consequently, the privacy and regulatory considerations of these solutions vary.

Even though **cloud computing** offers numerous technical benefits, such as extensive data storage, computational capabilities, and diverse services, it significantly lowers service costs and resolves resource limitations by efficiently sharing critical resources among multiple users [71]. However, in cloud computing environments, the security implications are particularly significant due to the dispersed storage of data across multiple international locations, where users lack physical access to their sensitive data, especially when data are outsourced to a third party [20]. Thus, before transferring AAL private and sensitive data to the cloud, it is essential to safeguard this information through various data protection methods. There are non-cryptographic techniques (such as data anonymisation, data splitting, and steganography) and cryptographic techniques, which are more widely used. These techniques have pros and cons. Cryptography offers strong security measures but can limit cloud functionalities and increase computation costs for handling cloud data. Data splitting reduces computation expenses but provides lower security levels. Data anonymisation includes perturbative masking, which alters data with dummy information, ensuring high security but limiting certain functionalities [20,71].

**Hybrid cloud** solutions enable transferring data between the public and private cloud, aiming to integrate various technologies for enhanced privacy protection and efficient cloud data management, thereby balancing data utilization with privacy [72]. However, transferring data to public cloud services introduces security and other challenges, including technical complexity in integrating diverse technologies, effective management practices, and efficient data search [73]. Transitioning from a public cloud to a hybrid cloud environment requires effective management to address the manageability challenges inherent in hybrid cloud infrastructure. Given that data owners and cloud storage services operate in different domains, the highly sensitive nature of data transferred within AAL systems makes encryption essential prior to outsourcing [74]. Nonetheless, encryption presents additional challenges, particularly in terms of searching and accessing data within the cloud environment, which complicates efficient data use [75].

**Figure 2:**
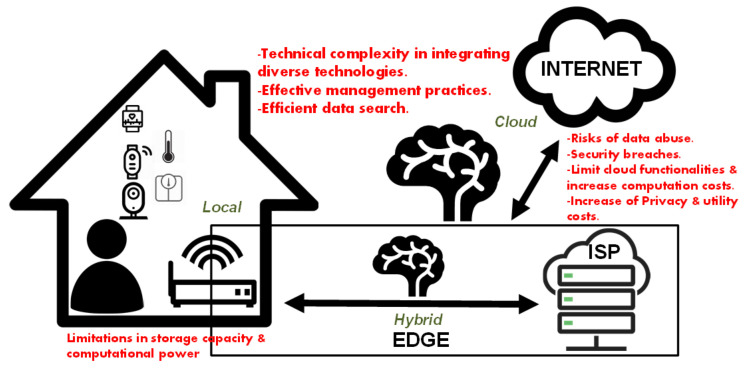
Data storage and processing approaches in sensor networks.

In contrast, **local data** processing provides dedicated resources, improving security and privacy. As data stays within the local environment, it is not transmitted over potentially insecure channels, reducing the risk of breaches and unauthorized access. Additionally, local processing lowers latency, offering faster response times. This is crucial for emergency healthcare situations, making it more reliable since it does not rely on internet connectivity or remote servers. However, local processing systems often face limitations in storage capacity and computational power, making it challenging to handle large volumes of data or complex analyses. Relying solely on local data processing may reduce the effectiveness of detection, monitoring, and prediction techniques by limiting the scope for population-based analysis. In AAL systems for elderly care, where the need for continuous data analysis is paramount, local processing architectures are employed to capitalize on their quick response times and robust data protection capabilities [69,70]. The current state of local data processing involves the use of edge computing devices, which bring computational power closer to the data source, such as edge-based AAL, rather than relying on centralized cloud servers [70]. This approach can mitigate some of the limitations by distributing processing tasks across multiple devices, yet it requires complicated management to ensure data consistency and effective resource utilization [76]. The Edge-based Assisted Living Platform (E-ALPHA) leverages a simulation-driven approach that supports both edge and cloud computing to optimize AAL service development across different scales. Such platforms enhance interoperability with IoT platforms and facilitate preliminary performance assessments to tailor services for real-world applications [70]. Balancing trade-offs between local and cloud processing remains a critical consideration for the design and implementation of sensor networks in healthcare. The key features of these three primary approaches for data storage and processing within sensor networks are summarized in Figure 2.

#### Communication Protocols

Real-time health-monitoring systems are essential for managing common age-related conditions such as diabetes, hypertension, heart disease, sleep apnea, cancer, etc. These systems utilize advanced communication protocols like ZigBee, LoRaWAN, and RFID to enable remote monitoring of patient health. For instance, LoRaWAN is good in long-range [77], low-power applications, making it ideal for rural healthcare settings, while ZigBee is more suitable for short-range communication [78] and demonstrates high accuracy in detecting patient location, achieving nearly 99% accuracy when compared with manual readings [79,80]. RFID also supports efficient data collection, particularly in environments with multiple monitored individuals. Generally managing network longevity with low-power devices becomes increasingly difficult as data volumes rise, impacting both accuracy and processing capacity. Additionally, the high costs of IoT implementation and maintenance pose a significant barrier, particularly for smaller healthcare providers [81,82,83].

### 3.2. Data and Communication Challenges and Trade-Offs

#### 3.2.1. Privacy, Security, and Data Management

Many off-the- shelf AAL devices rely on cloud-based systems for data transmission, storage, and processing, utilizing centralized servers for scalability and easier management.

In IoT–cloud-based e-Health frameworks, IoT networks facilitate communication among users, services, and systems, with clinical data being stored in the cloud [84]. IoT-based health-monitoring devices have demonstrated maximum relative errors of 2.89% in heart rate measurements, 3.03% in body temperature, and 1.05% in $SpO_{2}$ levels, comparable to commercial health-monitoring systems [34]. This cloud computing approach allows healthcare providers to respond more rapidly and accurately to patient data, although it introduces security risks [85].

High-profile breaches, such as those at Facebook [86] and Yahoo [87], highlight vulnerabilities that could lead to the theft or misuse of sensitive health data, which are highly valuable on illegal markets [88]. Cloud environments, often hosting multiple clients on shared infrastructure, create single points of failure and are susceptible to attacks, such as man-in-the-middle (MitM), during data transmissions. These risks require enhanced identity management, strict data control, and additional security measures, raising operational costs for medical service providers [89]. Furthermore, backup systems reliant on third-party providers raise additional concerns regarding data control and identity management. AAL sensors generate critical health data that are processed through aggregators using Bayesian or Markov probability models. These probabilistic models infer activities of daily living and detect early disease symptoms by identifying changes in routine behavior [90,91,92]. Unlike financial data, personal health data once acquired can maintain pertinence, while credit card details can be deactivated, emphasizing the need for robust post-attack recovery strategies.

In the IoT ecosystem, managing data acquisition, analysis, storage, and integration presents notable challenges that can impact system performance. Determining which data to retain and process locally on sensors is complex, especially given sensors’ memory limitations. Solutions such as data reduction, contraction, and sampling have been proposed to handle the large data volumes generated by AAL devices [93]. Edge and cloud computing are the primary approaches for data processing. Edge computing, which processes data locally, offers low latency, real-time responses, and increased bandwidth efficiency, as well as enhanced privacy, by reducing the amount of data sent to central servers. However, it also increases vulnerabilities to physical tampering and local cyber-attacks due to its distributed nature. In contrast, cloud computing models, like software as a service (SaaS), present challenges in security due to interdependencies between services, leading to inconsistencies in security protocols and difficulties in pinpointing accountability in case of breaches [89].

#### 3.2.2. Privacy and Data Quality

AAL systems handle extensive sensitive data, including medical records, behavioral insights, and genomic information. In many cases, users of wearable devices, Fitbit trackers, Apple Watches, and Garmin fitness bands do not have ownership of their data, which are typically stored by manufacturers and may be shared with third parties, including location and anonymised data [94]. Protecting these data requires robust frameworks like encryption and data anonymisation, along with transparent data-sharing policies that give users control over their information. However, complete anonymity remains challenging, as algorithms can re-identify individuals by linking biometric data from wearables, such as the Samsung Galaxy Watch, Polar heart rate monitors, and Whoop fitness bands, with digital traces, like behavioral activities and location, raising concerns about tracking and personality prediction [95,96,97]. Security breaches in healthcare are ever-increasing, highlighted by the American Medical Collection Agency (AMCA) breach, which compromised 24 million patient records from 2018 to 2019 [98,99]. Encryption protocols like AES, RSA, and TLS help to secure data transfers by ensuring confidentiality and data integrity; however, maintaining interoperability between diverse communication technologies is still a challenge. Additionally, high levels of encryption can slow down data processing, which may negatively impact the performance of real-time systems, particularly in emergency healthcare scenarios, where delays could affect patient outcomes [100].

**Figure 3:**
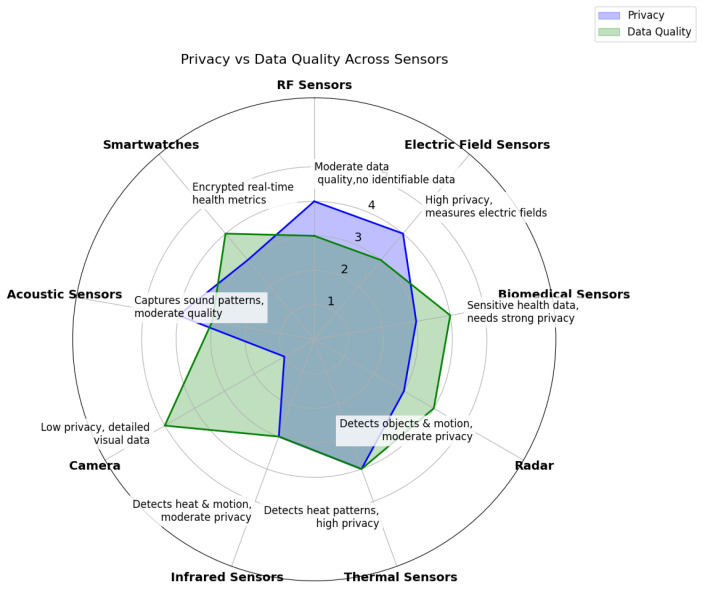
Privacy vs. data quality across AAL sensors (1 = Low, 5 = High).

Sensing technologies capture data by measuring physical, chemical, or biological parameters and convert them into signals for further analysis. However, data quality from sensors can vary significantly depending on the application and context, with accuracy, reliability, and relevance being key indicators of quality. Higher-quality data generally lead to better insights but also raise privacy concerns, as more detailed information increases the risk of exposing sensitive user data. Achieving a balance between data quality and privacy remains a core challenge in sensor-based applications. Comparative analyses of sensors must account for the qualitative differences in the data that they provide, as illustrated in Figure 3, which evaluates common healthcare sensors. Although, ideally, sensors would deliver high-quality data without compromising privacy, this is often difficult to achieve, requiring careful consideration of their suitability for home use. Notably, societal attitudes toward sensors and smart devices have evolved; for example, smart speakers with microphones, once seen as invasive, are now widely accepted. The ratings presented in Figure 3 reflect our interpretation of various sensor technologies in AAL systems, considering both their privacy implications and data quality levels. These assessments are informed by a combination of existing research, such as studies on sensor security and privacy (e.g., [94]), as well as practical considerations of how these technologies are deployed in real-world AAL settings. The privacy ratings account for factors such as the potential for identifying personal information, while the data quality ratings reflect the sensor’s ability to capture accurate and actionable information. While some sensors like cameras have a low privacy rating due to their ability to capture identifiable visual data, others like thermal sensors maintain a high level of privacy by detecting non-identifiable heat signatures. Similarly, biomedical sensors provide high data quality but require stringent privacy protections due to the sensitive nature of the health data that they collect.

## 4. Decision-Making Process

This section presents an overview of the decision-making methods used in AAL devices, emphasizing the challenges and opportunities associated with these approaches. It explores key trade-offs and challenges, particularly regarding cyber–physical security and the role of AI algorithms.

The objectives of the decision making in AAL systems center around enhancing user health, safety, and independence through activity recognition, pattern recognition, predictive analytics, and anomaly detection [7,101,102,103,104]. Addressing those objectives requires fusing data from multiple modalities to track daily activities, identify behavioral patterns, predict potential health risks, and detect anomalies that may signal emergencies [27]. Such anomalies may include falls, which have been extensively studied as a major cause of injuries and deaths for elderly individuals. Various video modalities, such as RGB, infrared, and thermal cameras, can be employed in home environments for fall detection. Anomaly detection frameworks, including autoencoders and their variations, are particularly useful for this purpose due to the data imbalance caused by the infrequent and diverse nature of falls [105]. However, the reliance on reconstruction errors in autoencoders can constrain the use of network architectures that propagate information effectively [106,107].

Seamless data fusion across diverse sources remains a challenge, especially as AAL environments are designed to meet the complex needs of elderly users who require consistent, unobtrusive monitoring and personalized care. To achieve the latter, some approaches employ modal decision-making systems that combine knowledge-based and non-knowledge-based subsystems, delivering targeted intelligence across those modalities to enhance system adaptability, decision accuracy, and more personalized services [7]. This knowledge system is generated based on policies answers (“what to do”) and user models (“who will act”).

By analyzing normal daily routines using patterns recognition, systems can detect deviations that may indicate health risks, such as the onset of cognitive decline, changes in mobility, or potential falls. Anomaly detection in this context benefits from sensor fusion, which integrates data from diverse sensors to provide a comprehensive understanding of a user’s condition, reducing false positives and improving the system’s ability to detect significant events in real time [23,108]. The integration of multiple modalities enhances signal accuracy by fusing redundant and complementary data from various sensors. This improves robustness and provides a more holistic analysis. However, such systems require careful optimization to balance performance, energy efficiency and privacy.

### 4.1. Decision-Making Challenges and Trade-Offs

One of the main features required by AAL systems is their ability to adapt and react in real time in response to dynamic changes, such as user interactions, sensor inputs, system component interactions, or emergencies. Adaptability and personalization are becoming more and more important, requiring scalability and flexibility throughout the system’s lifecycle, while also ensuring system requirements, like reliability, privacy, security and time of reaction, are maintained [109].

#### 4.1.1. Privacy

Privacy of control and monitoring algorithms has been explored in the control theory literature for cyber-physical systems [25,26,110]. These studies primarily focus on distributed architectures that require communication among spatially distributed agents [110]. However, healthcare applications have not been extensively considered. Privacy concerns revolve around anonymising or pseudonymising sensitive data, such as medical diagnoses, to protect individual identities. Privacy-preserving algorithms and access control mechanisms are implemented to maintain confidentiality while still allowing for effective decision support [111,112]. Challenges in this context include the difficulty in balancing data anonymisation with maintaining data utility for accurate decision making, particularly in healthcare. For example, if a healthcare provider anonymises patient data by removing specific health indicators, it can hinder researchers’ ability to identify trends or patterns critical for developing new treatments [110].

Common methods employed in the healthcare domain include **Differential Privacy**. Differential privacy is particularly advantageous due to its quantifiable mathematical characteristics, making it a valuable tool for addressing various privacy-related issues across multiple domains, especially in health monitoring [113,114]. The challenge lies in balancing the accuracy of detection with the level of privacy, which depends on the amount and type of noise added and the point in the data processing pipeline where it is applied. This method is particularly useful in applications requiring anomaly detection, such as detecting cyber-attacks in intelligent transportation systems (ITSs) or monitoring epidemic spread [115]. Recent studies on privacy-preserving controllers [110] have demonstrated that a high level of differential privacy can be achieved by introducing small noises.

Another proposed method consists of **Watermarking**, which embeds identifiers in signals or communicated packages to detect unauthorized access or data leaks [116]. This method can ensure data integrity and trace the source of the breach, making it a useful tool for maintaining privacy in networked systems. This is particularly useful in scenarios where data are distributed across various agents and the origin of data leaks needs to be identified. Research has used a dynamic version of watermarking as a solution to the problem of the secure control of cyber–physical systems, which inherently rely on multiple/sensors and actuators to interface with the physical environment and communicate over a network, acknowledging the possibility that some sensors may act maliciously by providing inaccurate or false measurements [117].

An interesting approach is represented by **Homomorphic** encryption, which allows computations on encrypted data but adds computational overhead [118]. It is particularly beneficial in cloud-based control systems, where data are often processed off-site [119]. For example, in smart grids, homomorphic encryption can be used to process consumption data without revealing individual household information [120]. Another approach is **Secure Multi-Party Computation** (SMPC), which enables joint computations without revealing individual inputs. This technique is useful in distributed optimization and control, where different agents work together without sharing their private data [121].

These developed methodologies allow one to remove the physical meaning from the communicated information, or to detect eavesdroppers. Challenges in these systems involve managing communication overhead, handling device heterogeneity, and ensuring scalability [122,123]. While specific research on privacy methodologies in AAL applications is limited, many techniques developed for cyber–physical systems (CPSs) can be adapted and extended for AAL environments.

#### 4.1.2. AI-Related Challenges

AAL devices serve as critical analytical tools, particularly when supported by extensive datasets from diverse sources [124]. Recent studies emphasize transitioning toward advanced, minimally invasive technologies that improve patient experience while aiding physicians in diagnosing and monitoring conditions efficiently [125]. This transition includes increased utilization of machine learning for disease detection and categorization, combined with AI-driven data analysis to support rapid interventions and a preventive, rather than reactive, healthcare model. AI holds transformative potential in disease prevention through predictive modeling, enabling the early detection of health threats by scrutinizing multiple data sources, like social media and electronic health records, which can expedite containment measures [126]. ML algorithms further enhance proactive care by recognizing disease patterns and risk factors, enabling tailored preventive measures [48].

AI-driven disease diagnosis can significantly reduce healthcare workloads, especially in remote settings; however, it encounters challenges in critical and emergency care, where gathering extensive clinical data for accurate AI models may be unfeasible [127]. The reliability of these models depends on high-quality, precise, and complete datasets for training [128]. Yet, many datasets in healthcare applications involve multi-modal, incomplete, wrongly labeled data, making most ML tasks challenging. Many classification algorithms assume identically distributed input data, which is often not satisfied in healthcare settings where human activities are interdependent and complex. Aggregating data from multiple healthcare centers can increase model accuracy by enriching data diversity [125], but may represent issues in terms of heterogeneous data and structure, as well as requiring stringent privacy protections. Techniques like federated learning enable distributed model training across multiple locations without centralizing raw data, updating models locally at each site to minimize privacy risks [129]. Further more, when utilizing healthcare data from multiple centers, ensuring patient privacy requires rigorous de-identification, aggregation, and anonymisation protocols to prevent misuse and safeguard sensitive information [130].

Ethical and legal ramifications of AI in healthcare, particularly around fairness and bias, are crucial concerns [131]. AI classifiers that exhibit disproportionate inaccuracies for minority groups can result in biased and unfair outcomes, raising questions about equitable treatment [132]. Nonetheless, some argue from a utilitarian perspective that AI systems with minor biases may still hold significant value if the overall benefit to public health is substantial [133]. Methodologies used to address bias in ML/AI-based tools are under investigation by a large part of the scientific ML community [134].

#### 4.1.3. Cyber–Physical Security

Cybersecurity poses a substantial challenge for decision making in AAL systems, as vulnerabilities in both cyber and physical layers can undermine the accuracy, reliability, and timeliness of critical interventions, thus having an effect in the physical world, possibly undermining the health and safety of the patient, as well as the functioning of the devices. Attacks at the physical layer, such as sensor tampering, can introduce false data, while communication layer threats like jamming or interception may delay or alter information, directly endangering user safety. Cyber intrusions in AAL systems can be categorized based on their impact: the first category targets data confidentiality, with unauthorized access to sensitive patient information that raises ethical concerns and risks identity theft or misuse of personal data; the second category aims to disrupt system functionality, including tampering with alarms or altering environmental settings crucial for patient safety, potentially causing medical equipment malfunctions or hazardous conditions [135,136]. A rising risk involves wearable and implantable medical devices (IMDs) vulnerable to hacking or tampering, which could not only impair device function but also affect patient’s safety [137,138]. These devices, often controlled via external care-link monitors, transmitters, or wireless protocols like WiFi, 5G, and Bluetooth, are vulnerable to various attack types, including the potential deactivation of critical alarms essential for prompt medical intervention [137,139].

Specific device cyber–physical vulnerabilities have also been investigated. For example, Medtronic’s implantable cardioverter defibrillators (ICDs) transmit operational data remotely [140] but lack encryption and authentication for critical functions, making them susceptible to unauthorized modifications of ICD parameters [141]. Similarly, Abbott’s pacemakers have firmware and communication vulnerabilities, allowing attackers to alter settings, deplete batteries, or deliver inappropriate shocks, potentially requiring surgical intervention or posing life-threatening risks [142].

## 5. AAL System Challenges and Trade-Offs

This section considers AAL frameworks as a system and highlights the interdisciplinary nature of addressing the open research challenges in AAL, which span across privacy, security, system performance and reliability, usability, and cost. These challenges cannot be effectively tackled by a single research community but demand a collaborative effort across fields, including sensing technologies, communication, and security and control systems, as well as considering the medical perspective. By examining these areas at the system level, we clarify the complex trade-offs involved in AAL system design and operation, particularly within healthcare applications.

### 5.1. Privacy and Security

#### 5.1.1. Privacy

The data lifecycle in IoT systems, from sensor deployment to data storage and the transformation of raw data into actionable insights, introduces privacy challenges at every stage [143]. These concerns extend beyond any single research domain but require a comprehensive approach to address the vulnerabilities that emerge throughout system layers, as highlighted in Section 3 and Section 4. The most critical areas are the communication and storage of data, where securing both of these is key to privacy. For example, vulnerabilities in the communication layer demonstrate the tension between excessive encryption, which enhances privacy, and the need for real-time decision-making accuracy.

Edge computing enables intelligent, real-time responses, which leads to faster data processing and reduced energy consumption [144,145]. It also minimizes the need to transmit sensitive health data over networks. However, this framework requires devices to have sufficient processing capacity and energy resources. If devices lack these, energy inefficiency can arise, and they may struggle to handle complex tasks like deep learning or large-scale data analysis in real time. Fog computing improves bandwidth efficiency by utilizing a larger network of devices. It reduces data transfer latency and complements cloud computing by lowering energy consumption and mitigating the risk of data overload [146]. However, it increases system complexity by adding more entities involved in data processing. This can expand the attack surface, making the system more vulnerable to cyber-threats.

While statistical summaries are often employed to protect privacy, advanced data mining techniques can still merge these summaries with external datasets, risking personal information disclosure [147]. Wearable devices, such as Fitbit trackers, Apple Watches, and Oura Rings, further complicate the issue, as data are frequently transmitted not only to researchers but also to companies and third-party entities involved in data collection, raising additional concerns, as seen with menstrual tracking apps selling user data to third parties, including government agencies [148].

Unlike more “open” technologies, such as Zigbee-based sensors (used in smart home systems for temperature and motion detection), which allow for communication-level openness but restrict access to internal firmware, most wearable devices are siloed. These wearables generally restrict customization or integration beyond their manufacturer-controlled platforms. For example, Fitbit offers cloud-based access to data via an API, but it limits what external applications can carry out with the data and requires authentication tokens. Similarly, Apple’s HealthKit and Oura’s Cloud API provide some data access but remain locked into specific ecosystems that require users to comply with proprietary terms and privacy policies.

On the other hand, “open” sensor platforms such as LoRaWAN (Long Range Wide Area Network) and Bluetooth Low Energy (BLE) sensors are often designed to be interoperable and support a broader range of devices and applications. Zigbee-based sensors, while restricted in terms of firmware modifications, enable integration with various smart home hubs (e.g., Amazon Echo or Google Home) and have open protocols for communication, which makes them more adaptable to third-party applications and devices. These open platforms provide the flexibility of integrating data from different manufacturers, offering users more control over their systems.

The risks associated with siloed technologies are further compounded in specific cases, such as menstrual tracking apps like Flo and Clue, which have been found selling user data to third parties, including government agencies [148]. These apps are typically part of proprietary ecosystems that limit the user’s ability to control or even access their own data.

Privacy concerns become even more pronounced in healthcare monitoring, particularly with invasive sensors like cameras. Less intrusive alternatives, such as radar-based sensors, are emerging as a promising solution that balances data collection efficiency with user privacy while ensuring compliance with healthcare regulations and minimizing deployment costs. Addressing privacy challenges demands an interdisciplinary approach that extends beyond sensor technologies. Vulnerabilities at the communication layer, for instance, highlight the trade-off between excessive encryption and real-time decision-making accuracy. Thus, solving these open research challenges requires collaboration across multiple disciplines, as no single area can tackle all privacy concerns in isolation.

#### 5.1.2. Security

Ensuring the security of health data is equally crucial for preventing unauthorized access, breaches, and misuse. Cybersecurity safeguards must encompass networks, hardware, systems, and data from digital threats [149]. Wearable devices, in particular, are susceptible to hacking due to the various communication technologies used to facilitate data transfer between the devices and smartphones [150]. Addressing these security challenges involves complex trade-offs among security, latency, and data processing, as protections like encryption can introduce delays that hinder real-time decision making. Ensuring effective cyber–physical security thus requires interdisciplinary coordination across sensors, communication protocols, and AI algorithms to guard against multi-layered attacks.

Federated Identity Management (FIM) protocols like OAuth [151] and Security Assertion Markup Language (SAML) [152] offer a streamlined approach to managing identities across numerous AAL devices, alleviating the administrative challenges of traditional Identity and Access Management (IAM) systems. These protocols provide a unified view of data that enhances the efficiency of healthcare services for both patients and professionals [153]. However, despite these advantages, FIM frameworks are also susceptible to several types of cyber-attacks, such as token theft, replay attacks, message insertion, and man-in-the-middle attacks.

Awareness of vulnerabilities inherent in AAL devices, spanning firmware, operating systems, data encryption, storage, and transmission, underscores the significant cyber risks confronting the present digital healthcare system. Combined vulnerabilities within the firmware integrity measurement (FIM) framework and AAL devices pose substantial cybersecurity threats to the current healthcare system. This entails a multifaceted assessment of vulnerabilities stemming from existing IT communication protocols, infrastructure, AAL and MCPS medical devices, FIM protocols, and their collective impact on hospital operations, accompanied by corresponding recommendations for security controls [153].

Effective AAL system deployment requires addressing privacy and security at both the design and operational stages to meet static and dynamic requirements. AAL systems may need to adjust their behavior in real time in response to dynamic changes detected. These changes can include end-user interactions, input from external hardware and sensors, interactions among system components, or the detection of emergency situations. These adaptive and dynamic properties must be managed at runtime, requiring dynamic modifications throughout the system’s lifecycle [109]. In this context, incorporating privacy and security “by design” establishes foundational protections preventing vulnerabilities from arising later during operation. Addressing dynamic security needs during the operational phase then enables systems to adapt to emerging threats and evolving data requirements. This proactive approach balances security, latency, and data processing, ensuring that AAL systems remain resilient and responsive throughout their lifecycle, thus reducing the need for reactive fixes. This approach requires interdisciplinary and holistic efforts.

#### 5.1.3. Regulatory Laws and Compliance

Regulatory laws for medical devices vary by region. However, common challenges exist in the EU, US, and UK. One key issue is the increased workload for notified bodies, which leads to delays in market entry. These processes are time-consuming and often require additional staff and expertise, slowing down compliance efforts [154]. The cost range for obtaining regulatory approval for medical devices varies based on the regulatory pathway, device classification, intended application, and whether the applicant qualifies as a small business. For an overview on these regulations and their challenges, see Table 1.

Furthermore, the lack of detailed regulatory guidance introduces uncertainty in translating requirements into technical specifications, making the process resource-intensive [155,156,157]. However, uncertified smart devices cannot inform healthcare decisions, limiting their utility until proper certification is obtained.

**Table 1:** Overview of regulatory approval, costs, performance metrics, and challenges for AAL devices.

Regulatory Approval and Key Regulatory Bodies	Device Type and Estimated Cost Range	Performance Metrics	Key Challenges and Requirements
**MDR CE (EU)** Medical Device Coordination Group (MDCG) [158,159]	Wearables, Sensors, Medical Devices Cost: EUR 5000–50,000 for Class I (low-risk, self-certification) to over EUR 150,000 for high-risk Class III devices.	Accuracy, Reliability, Data Integration, Long-term Safety	Stringent clinical trials, performance evaluations, and documentation required for market approval. High workload for notified bodies delays market entry. Additional costs for post-market surveillance.
**FDA (US)** Food and Drug Administration (FDA) [160]	Wearables, Sensors, Medical Devices Cost: USD 1000–10,000 for Class I (low-risk) devices to USD 2,000,000+ for high-risk devices.	Clinical Accuracy, Health Metrics	Requires premarket approval (PMA) or 510(k) clearance. High-risk devices demand extensive clinical evidence. Post-market surveillance emphasized through Medical Device Reporting (MDR) system.
**MHRA (UK)** Medicines and Healthcare products Regulatory Agency (MHRA) [161]	Wearables, Medical Devices Cost: GBP 1000–5000 for low-risk Class I devices and up to GBP 100,000+ for medium-risk devices.	Durability, Usability, Safety	Ensures compliance with performance standards and conducts regular audits. Challenges include meeting evolving regulations and maintaining continuous surveillance.
**ISO 13485 (Quality)** Global Standard [162]	Medical and Health-Monitoring Devices Cost: USD 5000–30,000	Durability, Usability, Safety	Focuses on quality system compliance, risk management, and operational effectiveness. Requires comprehensive documentation and compliance auditing, which can be resource-intensive.
**Data Privacy Standards** GDPR (EU) [163], HIPAA [164] (US)	Wearables, IoT Medical Devices Variable Cost	Data Integrity, Encryption	Compliance with regional data privacy laws. Challenges with cloud-based storage, patient consent, and ensuring data sovereignty.

To safeguard patients’ data, stringent regulations and severe penalties must be enforced by governments and healthcare organizations. However, most compliance standards do not address how to comply with legislation in the cloud computing. With data stored in the provider’s data centers, achieving compliance in sensing systems becomes complex. This complexity can result in issues related to regulatory compliance, such as data privacy, segregation, and security, which the provider must enforce [89].

### 5.2. Performance, Reliability, and Integration

It is challenging to optimize AAL systems overall due to the presence of multiple objectives spanning different dimensions and fields. AAL systems face several challenges in sensor integration and optimization, including sensor placement, fusion, and reliability in real-world settings. Optimizing sensor placement involves balancing technical capabilities with physical constraints, such as interference and space limitations, while also considering functional requirements. These objectives often conflict; for example, maximizing sensor coverage can increase energy consumption or reduce system privacy. Genetic algorithms have proven useful in addressing some of these challenges. For example, Soman et al. introduced the modal clarity index (MCI) to enhance sensor network performance, moving beyond traditional criteria, while Sun et al. extended this approach to optimize multi-sensor setups for improved damage detection [165,166,167].

These systems highlight the trade-offs between sensor accuracy and privacy preservation, particularly in continuous home monitoring scenarios. Similarly, sleep monitoring use cases demonstrate the application of non-invasive radar sensors and wearable devices to detect sleep positions and apnea. These systems effectively balance privacy concerns with the need for accurate physiological data collection, showcasing the feasibility of privacy-preserving AAL solutions. Furthermore, in tremor monitoring for Parkinson’s patients [168], wearables have been integrated with edge computing architectures to perform real-time analysis of tremor severity, providing insights into disease progression while maintaining data security [169,170].

Additionally, wearable devices offer real-time health monitoring but also face reliability challenges due to user engagement and long-term adoption [31,171]. Motion sensors have shown promise in fall detection and gait analysis, yet effective multi-sensor frameworks for comprehensive home-based gait analysis are still lacking [172,173]. Techniques such as RFID [174,175] and smartphone-based monitoring with inertial and geospatial capabilities have been explored [176,177], but their limitations in monitoring specific activities, such as household tasks, only capturing two feature domains (time and frequency), have led researchers to combine data from multiple sources for improved accuracy [178].

Camera-based sensors provide context, enhancing the accuracy of activity classification when combined with accelerometer data. Studies indicate that a single accelerometer may be insufficient, requiring the use of multiple sensors for more accurate results [39,40,178,179]. Video-based activity recognition, leveraging RGB and depth sensors, shows promise but faces difficulties in unstructured environments, where action recognition alone does not provide comprehensive analysis [180,181]. Moreover, inertial sensors can drift from their ideal positions during movements, potentially compromising data accuracy and quality. Their limited battery capacities also make them challenging for long-term monitoring [182].

The shift toward edge computing in AAL systems improves data reliability by maintaining operation during connectivity disruptions—see Section 4—but limitations in computational power and storage present trade-offs, potentially affecting detection accuracy and system resilience [89]. Long-term monitoring also faces challenges, as lab-tested solutions often fail to perform reliably in home settings due to environmental variability [183]. Additionally, the trade-off between data collection volume and device capabilities complicates critical event detection, with some wearable monitors displaying significant error margins of up to 25% [184,185]. Hence, balancing the above-mentioned trade-offs requires aligning optimization strategies with the system’s specific nature and priorities.

### 5.3. Cost

AAL technologies offer affordable, user-friendly solutions with various features, but the costs associated with these systems are varied, spanning financial, energy, and storage aspects.

#### 5.3.1. Financial

The cost of AAL solutions includes the acquisition of sensors and equipment, installation, and ongoing maintenance to ensure proper functionality. Operational expenses, such as electricity, network bandwidth, and consumables, also contribute to the total cost throughout the device lifecycle [186]. In healthcare, value-based reimbursement models, like those incentivized by MACRA, promote cost-effective care delivery. These models encourage the use of telehealth and remote monitoring systems, which help identify high-risk individuals and optimize integrated care teams [187]. Additionally, ensuring that assisted living technologies meet security, privacy, and safety standards requires rigorous testing and clinical evaluations, which further influence overall costs.

Most studies in this field primarily focus on reporting the cost of equipment [188], making it challenging to conduct an accurate comparative analysis of the overall cost or cost savings of AAL systems. These costs also vary significantly depending on the country, equipment, related health issue, and brand. However, industry statistics indicate substantial growth in the AAL market. Industry report Market Research Future estimates that the AAL market will be valued at USD 23.5 billion by 2030, with a CAGR of 19.1% during the forecast period [189]. In terms of cost savings, a study published in BMC Health Services Research reported that home care, a common application of AAL systems, was 32% less expensive than traditional hospital care [190,191,192]. The annual medical and healthcare expenses associated with falls among older adults amount to approximately USD 50 billion, with this cost projected to rise both within the United States and globally [193,194]. Implementing AAL systems for fall detection can lead to significant cost savings by reducing fall-related incidents and enhancing response times. A study published in Sustainability in 2023 introduced a low-cost, motion-based technique for detecting falls among the elderly. This system utilized pyroelectric infrared sensors and achieved nearly 99% accuracy in fall detection, highlighting its potential for cost-effective fall monitoring [195]. Additionally, a report from McKnight’s Senior Living estimated that implementing fall detection wearable devices in senior living communities led to cost savings of approximately USD 21,068. These savings were primarily due to the fall prevention and improved staff response times [196].

#### 5.3.2. Energy Consumption, Storage, and AI

The longevity of sensor networks depends heavily on the efficient management of energy consumption across their sensing, processing, and communication components. To maximize the lifespan of a wireless sensor network (WSN), it is crucial to select low-power radio technologies [197,198] and implement energy-efficient network protocols and messaging strategies [17]. Additionally energy-aware routing, which involves utilizing energy-efficient routing protocols to extend the lifespan of resource-constrained devices, is major for preserving the energy of network nodes. Without these measures, excessive relaying can lead to premature battery depletion, diminishing network efficiency [199]. Energy consumption impacts not only the battery life of portable devices but also operational costs in larger installations, such as increased electricity bills and the frequent need for battery replacements in wireless sensors [200]. This is particularly significant for smart healthcare systems, which rely on wearable devices and smartphones for long-term patient monitoring. Addressing these challenges involves using long-lasting batteries [201], low-power hardware components [202], and innovative energy harvesting techniques [203]. Moreover, incorporating sensors with ‘sleep’ and `wake-up’ functionalities optimizes energy usage without compromising system performance [204].

AI-powered sensors and models require not only energy for training but also for ongoing inference, which occurs whenever the model processes real-time data. To address this, some researchers and companies are exploring ways to improve the energy efficiency of AI, such as optimizing data center operations, utilizing energy-efficient hardware, and creating frameworks that allow AI developers to monitor and reduce their carbon footprint [205]. These solutions are particularly important for healthcare applications, yet energy resources can be limited [206,207]. Reducing these energy and financial costs requires a comprehensive understanding of the system’s overall requirements at both the communication and decision-making levels.

### 5.4. Usability and User Friendliness

Studies on consumer behavior and opinion toward smart wearable devices have explored key technical and psychological factors, often applying extended versions of the technology acceptance model (TAM) [208]. Most research focuses on initial adoption rather than long-term usage, with few studies examining sustained engagement [209]. Ethical transparency and user education on privacy are also crucial considerations, making the adoption of wearables in healthcare settings a complex issue [210]. Users may resist or be reluctant to adopt monitoring technology due to unfamiliar human–machine interfaces, feelings of intrusiveness, or discomfort. The impact on quality of life can include inconvenience, stress, or anxiety from constant monitoring, which must be considered in the overall cost assessment [209].

Usability studies often fail to test devices with the target population, overlooking unique user needs. For instance, 32% of users stop using wearable devices within six months, and 50% within a year, citing concerns about safety, reliability, and accuracy [211,212]. Older adults, in particular, struggle with device interfaces and installation processes, exacerbated by sensory impairments. Moreover, while smartphones are frequently used to connect with wearables, the lack of companion apps limits users’ ability to analyze and visualize collected data, and many designs neglect critical usability aspects [212]. Addressing these usability challenges requires a comprehensive understanding of both system functionality and non-functional requirements, which must be considered during design and operational phases.

## 6. Conclusions

This article provides an overview of current and emerging AAL solutions, focusing on three key domains: sensors, communications, and decision making. Designing efficient, reliable, and secure AAL systems requires a systems-level perspective. Hence, this article explores the interdisciplinary challenges that emerge when developing such solutions, including themes such as privacy/performance/cost/usability. It discusses where real-time processing is energy-efficient and secure without sacrificing the system’s timeliness or accuracy. Strategies like edge computing for immediate analysis and cloud storage for long-term trends, supported by AI algorithms to prioritize health data, can optimize data management and ensure timely interventions’ efficiency without compromising on privacy or efficiency.

Future research must prioritize the standardization of protocols and ensure interoperability across healthcare platforms while addressing key issues such as data ownership and privacy. Developing clear frameworks in these areas will be critical to optimizing the use of AAL systems, reducing healthcare costs, and enhancing patient-centered care. Due to the interconnected nature of these challenges, a holistic approach, involving collaboration across disciplines such as IoT, sensing, security, AI, and control systems, is essential for addressing both technical and ethical concerns, thereby facilitating the wider adoption of effective healthcare solutions. In this context, software engineering methodologies can support the integration of privacy and performance requirements throughout the system development lifecycle. Additionally, experimental validation will play a vital role in assessing the effectiveness of proposed solutions in real-world settings. Our ongoing work, which takes a holistic view of these challenges, is guiding the development of a novel interdisciplinary methodology. We are preparing to publish this methodology soon, with the aim of advancing both research and practical implementations in AAL.

## Data Availability

No new data were created or analyzed in this study. Data sharing is not applicable to this article.

## References

[1] Consel C., Jeffrey A. (2019). Aging with the Internet of Things. Bridge.

[2] Zhou H., Hu H. (2007). Inertial sensors for motion detection of human upper limbs. Sens. Rev..

[3] Yan T., Wang Z., Pan Z.J. (2018). Flexible strain sensors fabricated using carbon-based nanomaterials: A review. Curr. Opin. Solid State Mater. Sci..

[4] Igual R., Medrano C., Plaza I. (2013). Challenges, issues and trends in fall detection systems. Biomed. Eng. Online.

[5] Cardenas J.D., Gutierrez C.A., Aguilar-Ponce R. (2023). Deep learning multi-class approach for human fall detection based on doppler signatures. Int. J. Environ. Res. Public Health.

[6] Marco A., Casas R., Falco J., Gracia H., Artigas J.I., Roy A. (2008). Location-based services for elderly and disabled people. Comput. Commun..

[7] Panagiotou C., Panagiotakopoulos T., Kameas A. A multi: Modal Decision Making System for an Ambient Assisted Living Environment. Proceedings of the 8th ACM International Conference on Pervasive Technologies Related to Assistive Environments.

[8] Blasco R., Marco Á., Casas R., Cirujano D., Picking R. (2014). A smart kitchen for ambient assisted living. Sensors.

[9] Kim J., Campbell A.S., de Ávila B.E.F., Wang J. (2019). Wearable biosensors for healthcare monitoring. Nat. Biotechnol..

[10] Karimi-Maleh H., Orooji Y., Karimi F., Alizadeh M., Baghayeri M., Rouhi J., Tajik S., Beitollahi H., Agarwal S., Gupta V.K. (2021). A critical review on the use of potentiometric based biosensors for biomarkers detection. Biosens. Bioelectron..

[11] Krishnan S.K., Singh E., Singh P., Meyyappan M., Nalwa H.S. (2019). A review on graphene-based nanocomposites for electrochemical and fluorescent biosensors. RSC Adv..

[12] Aldeer M., Javanmard M., Martin R.P. (2018). A review of medication adherence monitoring technologies. Appl. Syst. Innov..

[13] Ahmad I., Asghar Z., Kumar T., Li G., Manzoor A., Mikhaylov K., Shah S.A., Höyhtyä M., Reponen J., Huusko J. (2022). Emerging technologies for next generation remote health care and assisted living. IEEE Access.

[14] Shetti N.P., Bukkitgar S.D., Reddy K.R., Reddy C.V., Aminabhavi T.M. (2019). ZnO-based nanostructured electrodes for electrochemical sensors and biosensors in biomedical applications. Biosens. Bioelectron..

[15] Wang J., Bauer J., Becker M., Bente P., Dasenbrock L., Elbers K., Hein A., Kohlmann M., Kolb G., Lammel-Polchau C. (2014). A novel approach for discovering human behavior patterns using unsupervised methods. Z. Gerontol. Geriatr..

[16] Wang J., Spicher N., Warnecke J.M., Haghi M., Schwartze J., Deserno T.M. (2021). Unobtrusive health monitoring in private spaces: The smart home. Sensors.

[17] McGrath M.J., Scanaill C.N. (2013). Sensor Technologies: Healthcare, Wellness, and Environmental Applications.

[18] Addante F., Gaetani F., Patrono L., Sancarlo D., Sergi I., Vergari G. (2019). An innovative AAL system based on IoT technologies for patients with sarcopenia. Sensors.

[19] Landi F., Liperoti R., Russo A., Giovannini S., Tosato M., Capoluongo E., Bernabei R., Onder G. (2012). Sarcopenia as a risk factor for falls in elderly individuals: Results from the ilSIRENTE study. Clin. Nutr..

[20] Xiao Z., Xiao Y. (2012). Security and privacy in cloud computing. IEEE Commun. Surv. Tutor..

[21] Dasios A., Gavalas D., Pantziou G., Konstantopoulos C. (2015). Hands-on experiences in deploying cost-effective ambient-assisted living systems. Sensors.

[22] Tunca C., Alemdar H., Ertan H., Incel O.D., Ersoy C. (2014). Multimodal wireless sensor network-based ambient assisted living in real homes with multiple residents. Sensors.

[23] Patel A., Shah J. (2019). Sensor-based activity recognition in the context of ambient assisted living systems: A review. J. Ambient Intell. Smart Environ..

[24] Sadek I., Rehman S.U., Codjo J., Abdulrazak B. (2019). Privacy and Security of IoT Based Healthcare Systems: Concerns, Solutions, and Recommendations. How AI Impacts Urban Living and Public Health, Proceedings of the 17th International Conference, ICOST 2019, New York City, NY, USA, 14–16 October 2019.

[25] Annaswamy A.M., Johansson K.H., Pappas G. (2024). Control for societal-scale challenges: Road map 2030. IEEE Control Syst. Mag..

[26] Lamnabhi-Lagarrigue F., Annaswamy A., Engell S., Isaksson A., Khargonekar P., Murray R.M., Nijmeijer H., Samad T., Tilbury D., Van den Hof P. (2017). Systems & control for the future of humanity, research agenda: Current and future roles, impact and grand challenges. Annu. Rev. Control.

[27] Calvaresi D., Cesarini D., Sernani P., Marinoni M., Dragoni A.F., Sturm A. (2017). Exploring the ambient assisted living domain: A systematic review. J. Ambient Intell. Humaniz. Comput..

[28] Rashidi P., Mihailidis A. (2012). A survey on ambient-assisted living tools for older adults. IEEE J. Biomed. Health Inform..

[29] Cicirelli G., Marani R., Petitti A., Milella A., D’Orazio T. (2021). Ambient assisted living: A review of technologies, methodologies and future perspectives for healthy aging of population. Sensors.

[30] Li R., Lu B., McDonald-Maier K.D. (2015). Cognitive assisted living ambient system: A survey. Digit. Commun. Netw..

[31] Yusif S., Soar J., Hafeez-Baig A. (2016). Older people, assistive technologies, and the barriers to adoption: A systematic review. Int. J. Med. Inform..

[32] Vimarlund V., Borycki E.M., Kushniruk A.W., Avenberg K. (2021). Ambient assisted living: Identifying new challenges and needs for digital technologies and service innovation. Yearb. Med. Inform..

[33] Caballero P., Ortiz G., Medina-Bulo I. (2024). Systematic literature review of ambient assisted living systems supported by the Internet of Things. Univers. Access Inf. Soc..

[34] Abdulmalek S., Nasir A., Jabbar W.A., Almuhaya M.A., Bairagi A.K., Khan M.A.M., Kee S.H. (2022). IoT-based healthcare-monitoring system towards improving quality of life: A review. Healthcare.

[35] Choukou M.A., Polyvyana A., Sakamoto Y., Osterreicher A. (2021). Ambient assisted living technologies to support older adults’ health and wellness: A systematic mapping review. Eur. Rev. Med. Pharmacol. Sci..

[36] Dimitrievski A., Zdravevski E., Lameski P., Trajkovik V. A Survey of Ambient Assisted Living Systems: Challenges and Opportunities. Proceedings of the 2016 IEEE 12th International Conference on Intelligent Computer Communication and Processing (ICCP).

[37] Javaid M., Haleem A., Rab S., Singh R.P., Suman R. (2021). Sensors for daily life: A review. Sens. Int..

[38] Postolache O.A., Mukhopadhyay S.C., Jayasundera K.P., Swain A.K. (2017). Sensors for Everyday Life.

[39] Nam Y., Rho S., Lee C. (2013). Physical activity recognition using multiple sensors embedded in a wearable device. ACM Trans. Embed. Comput. Syst..

[40] Hafeez S., Jalal A., Kamal S. Multi-Fusion Sensors for Action Recognition Based on Discriminative Motion Cues and Random Forest. Proceedings of the 2021 International Conference on Communication Technologies (ComTech).

[41] Broer A.A., Benedictus R., Zarouchas D. (2022). The need for multi-sensor data fusion in structural health monitoring of composite aircraft structures. Aerospace.

[42] Bartels J.H., Potthast T., Möller S., Grießmann T., Rolfes R., Beer M., Marx S. (2024). Robust SHM: Redundancy approach with different sensor integration levels for long life monitoring systems. e-J. Nondestruct. Test..

[43] Sood K., Nosouhi M.R., Kumar N., Gaddam A., Feng B., Yu S. (2021). Accurate detection of IoT sensor behaviors in legitimate, faulty and compromised scenarios. IEEE Trans. Dependable Secur. Comput..

[44] Beltrán J., Guindel C., De La Escalera A., García F. (2022). Automatic extrinsic calibration method for lidar and camera sensor setups. IEEE Trans. Intell. Transp. Syst..

[45] Taraldsen K., Chastin S.F., Riphagen I.I., Vereijken B., Helbostad J.L. (2012). Physical activity monitoring by use of accelerometer-based body-worn sensors in older adults: A systematic literature review of current knowledge and applications. Maturitas.

[46] Wu H. (2024). Multiscale entropy with electrocardiograph, electromyography, electroencephalography, and photoplethysmography signals in healthcare: A twelve-year systematic review. Biomed. Signal Process. Control.

[47] Middleton C. (2010). Delivering services over next generation broadband networks: Exploring devices, applications and networks. Telecommun. J. Aust..

[48] Palavicini G. (2023). Intelligent Health: Progress and Benefit of Artificial Intelligence in Sensing-Based Monitoring and Disease Diagnosis. Sensors.

[49] Denis S., Berkvens R., Weyn M. (2019). A survey on detection, tracking and identification in radio frequency-based device-free localization. Sensors.

[50] Chalmers C., Fergus P., Montanez C.A.C., Sikdar S., Ball F., Kendall B. (2020). Detecting activities of daily living and routine behaviours in dementia patients living alone using smart meter load disaggregation. IEEE Trans. Emerg. Top. Comput..

[51] Devlin M.A., Hayes B.P. (2019). Non-intrusive load monitoring and classification of activities of daily living using residential smart meter data. IEEE Trans. Consum. Electron..

[52] Klemenjak C., Makonin S., Elmenreich W. Towards Comparability in Non-Intrusive Load Monitoring: On Data and Performance Evaluation. Proceedings of the 2020 IEEE Power & Energy Society Innovative Smart Grid Technologies Conference (ISGT).

[53] Kelly J., Knottenbelt W. Neural Nilm: Deep Neural Networks Applied to Energy Disaggregation. Proceedings of the 2nd ACM International Conference on Embedded Systems for Energy-Efficient Built Environments.

[54] Sasaki Y. (2021). A survey on IoT big data analytic systems: Current and future. IEEE Internet Things J..

[55] Alabdulatif A., Khalil I., Forkan A.R.M., Atiquzzaman M. (2018). Real-time secure health surveillance for smarter health communities. IEEE Commun. Mag..

[56] Natgunanathan I., Mehmood A., Xiang Y., Gao L., Yu S. (2018). Location privacy protection in smart health care system. IEEE Internet Things J..

[57] Gupta R., Tanwar S., Tyagi S., Kumar N. (2020). Machine learning models for secure data analytics: A taxonomy and threat model. Comput. Commun..

[58] Goshvarpour A., Abbasi A., Goshvarpour A. (2017). An accurate emotion recognition system using ECG and GSR signals and matching pursuit method. Biomed. J..

[59] Catherwood P.A., Steele D., Little M., Mccomb S., McLaughlin J. (2018). A community-based IoT personalized wireless healthcare solution trial. IEEE J. Transl. Eng. Health Med..

[60] Prerna D., Tekchandani R., Kumar N. (2020). Device-to-device content caching techniques in 5G: A taxonomy, solutions, and challenges. Comput. Commun..

[61] Ren Y., Wang C., Chen Y., Yang J., Li H. (2019). Noninvasive fine-grained sleep monitoring leveraging smartphones. IEEE Internet Things J..

[62] Liu L., Xu J., Huan Y., Zou Z., Yeh S.C., Zheng L.R. (2019). A smart dental health-IoT platform based on intelligent hardware, deep learning, and mobile terminal. IEEE J. Biomed. Health Inform..

[63] Zuo Z., Watson M., Budgen D., Hall R., Kennelly C., Al Moubayed N. (2021). Data anonymisation for pervasive health care: Systematic literature mapping study. JMIR Med. Inform..

[64] Zhu H., Liu M., Fang C., Deng R., Cheng P. (2023). Detection-performance tradeoff for watermarking in industrial control systems. IEEE Trans. Inf. Forensics Secur..

[65] Brown E.A. (2016). The Fitbit fault line: Two proposals to protect health and fitness data at work. Yale J. Health Policy Law Ethics.

[66] Mhatre V., Rosenberg C. Homogeneous vs. Heterogeneous Clustered Sensor Networks: A comparative study. Proceedings of the 2004 IEEE International Conference on Communications (IEEE Cat. No. 04CH37577).

[67] Khriji S., Benbelgacem Y., Chéour R., Houssaini D.E., Kanoun O. (2022). Design and implementation of a cloud-based event-driven architecture for real-time data processing in wireless sensor networks. J. Supercomput..

[68] Alzahrani A., Alyas T., Alissa K., Abbas Q., Alsaawy Y., Tabassum N. (2022). Hybrid approach for improving the performance of data reliability in cloud storage management. Sensors.

[69] Wang K., Shao Y., Shu L., Han G., Zhu C. (2015). LDPA: A local data processing architecture in ambient assisted living communications. IEEE Commun. Mag..

[70] Aloi G., Fortino G., Gravina R., Pace P., Savaglio C. (2020). Simulation-driven platform for Edge-based AAL systems. IEEE J. Sel. Areas Commun..

[71] Khan A.A., Laghari A.A., Awan S., Jumani A.K. (2021). Fourth Industrial Revolution Application: Network Forensics Cloud Security Issues. Security Issues and Privacy Concerns in Industry 4.0 Applications.

[72] Yang J.J., Li J.Q., Niu Y. (2015). A hybrid solution for privacy preserving medical data sharing in the cloud environment. Future Gener. Comput. Syst..

[73] Khan S.U., Ullah N. (2016). Challenges in the adoption of hybrid cloud: An exploratory study using systematic literature review. J. Eng..

[74] Yan S., Chen C., Zhao G., Lee B.S. Cloud Service Recommendation and Selection for Enterprises. Proceedings of the 2012 8th iNternational Conference on Network and Service Management (cnsm) and 2012 Workshop on Systems Virtualiztion Management (svm).

[75] Li J., Li J., Chen X., Jia C., Liu Z. (2012). Efficient Keyword Search Over Encrypted Data with Fine-Grained Access Control in Hybrid Cloud. Network and System Security, Proceedings of the 6th International Conference, NSS 2012, Wuyishan, China, 21–23 November 2012.

[76] Naresh V.S., Pericherla S.S., Murty P.S.R., Reddi S. (2020). Internet of Things in Healthcare: Architecture, Applications, Challenges, and Solutions. Comput. Syst. Sci. Eng..

[77] Fernandes C.D., Depari A., Sisinni E., Ferrari P., Flammini A., Rinaldi S., Pasetti M. Hybrid Indoor and Outdoor Localization for Elderly Care Applications with LoRaWAN. Proceedings of the 2020 IEEE International Symposium on Medical Measurements and Applications (MeMeA).

[78] Vineetha Y., Misra Y., Kishore K.K. (2020). A real time IoT based patient health monitoring system using machine learning algorithms. Eur. J. Mol. Clin. Med.

[79] Hiraguri T., Aoyagi M., Morino Y., Akimoto T., Nishimori K., Hiraguri T. (2015). Proposal of ZigBee systems for the provision of location information and transmission of sensor data in medical welfare. E-Health Telecommun. Syst. Netw..

[80] Alwan O.S., Prahald Rao K. (2017). Dedicated real-time monitoring system for health care using ZigBee. Healthc. Technol. Lett..

[81] Khan M.A.A., Kabir M.A. (2016). Comparison among short range wireless networks: Bluetooth, Zig Bee & Wi-Fi. Daffodil Int. Univ. J. Sci. Technol..

[82] Kumar P., Silambarasan K. (2022). Enhancing the performance of healthcare service in IoT and cloud using optimized techniques. IETE J. Res..

[83] Verma N., Singh S., Prasad D. (2022). A review on existing IoT architecture and communication protocols used in healthcare monitoring system. J. Inst. Eng. Ser. B.

[84] Abdulghani H.A., Nijdam N.A., Collen A., Konstantas D. (2019). A study on security and privacy guidelines, countermeasures, threats: IoT data at rest perspective. Symmetry.

[85] Sivan R., Zukarnain Z.A. (2021). Security and privacy in cloud-based e-health system. Symmetry.

[86] Hutchinson B. Million Facebook Users to Find Out if Their Personal Data Was Breached, 87. https://abcnews.go.com/US/87-million-facebook-users-find-personal-data-breached/story?id=54334187.

[87] Garun N. (2017). Yahoo Says All 3 Billion User Accounts Were Impacted by 2013 Security Breach. https://www.nytimes.com/2017/10/03/technology/yahoo-hack-3-billion-users.html.

[88] Yao M. (2017). Your Electronic Medical Records Could Be Worth $1000 to Hackers. Forbes..

[89] Srivastava A., Singh P. (2022). Security Issues in Cloud Computing. J. Manag. Serv. Sci..

[90] Ullah I., Adhikari D., Su X., Palmieri F., Wu C., Choi C. (2024). Integration of data science with the intelligent IoT (IIoT): Current challenges and future perspectives. Digit. Commun. Netw..

[91] Seixas F.L., Zadrozny B., Laks J., Conci A., Saade D.C.M. (2014). A Bayesian network decision model for supporting the diagnosis of dementia, Alzheimer disease and mild cognitive impairment. Comput. Biol. Med..

[92] Cheng B.C., Tsai Y.A., Liao G.T., Byeon E.S. (2010). HMM machine learning and inference for activities of daily living recognition. J. Supercomput..

[93] Reichman O.J., Jones M.B., Schildhauer M.P. (2011). Challenges and opportunities of open data in ecology. Science.

[94] Venkataramanan M. (2014). My Identity for Sale. WIRED Magazine.

[95] De Montjoye Y.A., Hidalgo C.A., Verleysen M., Blondel V.D. (2013). Unique in the crowd: The privacy bounds of human mobility. Sci. Rep..

[96] Kosinski M., Stillwell D., Graepel T. (2013). Private traits and attributes are predictable from digital records of human behavior. Proc. Natl. Acad. Sci. USA.

[97] Lambiotte R., Kosinski M. (2014). Tracking the digital footprints of personality. Proc. IEEE.

[98] Seh A.H., Zarour M., Alenezi M., Sarkar A.K., Agrawal A., Kumar R., Ahmad Khan R. (2020). Healthcare data breaches: Insights and implications. Healthcare.

[99] Shachmurove N.C., McCulloch W. (2021). Health care companies face financial strain from data breaches. Am. Bankruptcy Inst. J..

[100] Shojaei P., Vlahu-Gjorgievska E., Chow Y.W. (2024). Security and privacy of technologies in health information systems: A systematic literature review. Computers.

[101] Moore D.H., Algase D.L., Powell-Cope G., Applegarth S., Beattie E.R. (2009). A framework for managing wandering and preventing elopement. Am. J. Alzheimer’s Dis. Other Dementias.

[102] Hein A., Kirste T. (2008). Activity Recognition for Ambient Assisted Living: Potential and Challenges. Ambient Assisted Living.

[103] Bao L., Intille S.S. Activity Recognition from User-Annotated Acceleration Data. Proceedings of the Conference on Pervasive Computing.

[104] Gulati N., Kaur P.D. (2021). An argumentation enabled decision making approach for Fall Activity Recognition in Social IoT based Ambient Assisted Living systems. Future Gener. Comput. Syst..

[105] Mishra P.K., Iaboni A., Ye B., Newman K., Mihailidis A., Khan S.S. (2023). Privacy-protecting behaviours of risk detection in people with dementia using videos. BioMed. Eng. OnLine.

[106] Mohan D., Al-Hamid D.Z., Chong P.H.J., Sudheera K.L.K., Gutierrez J., Chan H.C., Li H. (2024). Artificial Intelligence and IoT in Elderly Fall Prevention: A Review. IEEE Sens. J..

[107] Denkovski S., Khan S.S., Mihailidis A. Temporal Shift-Multi-Objective Loss Function for Improved Anomaly Fall Detection. Proceedings of the Asian Conference on Machine Learning, PMLR.

[108] Deep S., Zheng X., Karmakar C., Yu D., Hamey L.G., Jin J. (2019). A survey on anomalous behavior detection for elderly care using dense-sensing networks. IEEE Commun. Surv. Tutorials.

[109] Garcés L., Oquendo F., Nakagawa E.Y. A Quality Model for AAL Software Systems. Proceedings of the 2016 IEEE 29th International Symposium on Computer-Based Medical Systems (CBMS).

[110] Kawano Y., Cao M. (2020). Design of privacy-preserving dynamic controllers. IEEE Trans. Autom. Control.

[111] Zakka V.G., Dai Z., Manso L.J. Action Recognition for Privacy-Preserving Ambient Assisted Living. Proceedings of the International Conference on AI in Healthcare.

[112] Climent-Pérez P., Florez-Revuelta F. (2022). Privacy-preserving human action recognition with a many-objective evolutionary algorithm. Sensors.

[113] Dankar F.K., El Emam K. The Application of Differential Privacy to Health Data. Proceedings of the 2012 Joint EDBT/ICDT Workshops.

[114] Dankar F.K., El Emam K. (2013). Practicing differential privacy in health care: A review. Trans. Data Priv..

[115] Ferrari R.M., Teixeira A.M. (2021). Safety, Security and Privacy for Cyber-Physical Systems.

[116] Rubio-Hernan J., De Cicco L., Garcia-Alfaro J. (2017). On the use of watermark-based schemes to detect cyber-physical attacks. EURASIP J. Inf. Secur..

[117] Satchidanandan B., Kumar P.R. (2016). Dynamic watermarking: Active defense of networked cyber–physical systems. Proc. IEEE.

[118] Kogiso K., Fujita T. Cyber-Security Enhancement of Networked Control Systems Using Homomorphic Encryption. Proceedings of the 2015 54th IEEE Conference on Decision and Control (CDC).

[119] Zhou L., Varadharajan V., Hitchens M. (2013). Achieving secure role-based access control on encrypted data in cloud storage. IEEE Trans. Inf. Forensics Secur..

[120] Li F., Luo B., Liu P. Secure Information Aggregation for Smart Grids Using Homomorphic Encryption. Proceedings of the 2010 First IEEE International Conference on Smart Grid Communications.

[121] Si F., Zhang N., Wang Y., Kong P.Y., Qiao W. (2022). Distributed optimization for integrated energy systems with secure multiparty computation. IEEE Internet Things J..

[122] Barboni A. (2020). Diagnosis of Stealthy Local Cyber-Attacks in Large-Scale Systems. Ph.D. Thesis.

[123] Chen Z., Pasqualetti F., He J., Cheng P., Trentelman H.L., Bullo F. (2020). Guest editorial: Special issue on security and privacy of distributed algorithms and network systems. IEEE Trans. Autom. Control.

[124] Mohan V., Anand H. (2019). Biosensor: An emerging analytical tool. J. Biosens. Bioelectron.

[125] Olawade D.B., Wada O.J., David-Olawade A.C., Kunonga E., Abaire O., Ling J. (2023). Using artificial intelligence to improve public health: A narrative review. Front. Public Health.

[126] Lee D., Yoon S.N. (2021). Application of artificial intelligence-based technologies in the healthcare industry: Opportunities and challenges. Int. J. Environ. Res. Public Health.

[127] Erion G., Janizek J.D., Hudelson C., Utarnachitt R.B., McCoy A.M., Sayre M.R., White N.J., Lee S.I. (2022). A cost-aware framework for the development of AI models for healthcare applications. Nat. Biomed. Eng..

[128] Price W.N., Cohen I.G. (2019). Privacy in the age of medical big data. Nat. Med..

[129] Sheller M.J., Edwards B., Reina G.A., Martin J., Pati S., Kotrotsou A., Milchenko M., Xu W., Marcus D., Colen R.R. (2020). Federated learning in medicine: Facilitating multi-institutional collaborations without sharing patient data. Sci. Rep..

[130] Edemekong P.F., Annamaraju P., Haydel M.J. (2018). Health Insurance Portability and Accountability Act. https://pubmed.ncbi.nlm.nih.gov/29763195.

[131] Rajkomar A., Hardt M., Howell M.D., Corrado G., Chin M.H. (2018). Ensuring fairness in machine learning to advance health equity. Ann. Intern. Med..

[132] Hardt M., How Big Data is Unfair (2014). Medium. https://medium.com/@mrtz/how-big-data-is-unfair-9aa544d739de.

[133] Schönberger D. (2019). Artificial intelligence in healthcare: A critical analysis of the legal and ethical implications. Int. J. Law Inf. Technol..

[134] Gianfrancesco M.A., Tamang S., Yazdany J., Schmajuk G. (2018). Potential biases in machine learning algorithms using electronic health record data. JAMA Intern. Med..

[135] Ebong O., Edet A., Uwah A., Udoetor N. (2024). Comprehensive Impact Assessment of Intrusion Detection and Mitigation Strategies Using Support Vector Machine Classification. Res. J. Pure Sci. Technol..

[136] Buczak A.L., Guven E. (2015). A survey of data mining and machine learning methods for cyber security intrusion detection. IEEE Commun. Surv. Tutorials.

[137] Alexander B., Neira V., Campbell D., Crystal E., Simpson C., Enriquez A., Chacko S., Abdollah H., Redfearn D., Baranchuk A. (2020). Implantable Cardioverter-Defibrillator–Cybersecurity. Circ. Arrhythmia Electrophysiol..

[138] Alhussan M., Boem F., Ghoreishizadeh S.S., Mandalari A.M. From Eavesdropping to Exploitation: Exposing Vulnerabilities in BLE-Enabled Wearable Medical Devices. Proceedings of the EWSN, The 21st International Conference on Embedded Wireless Systems and Networks.

[139] Patel B., Makaryus A.N. (2021). Cardiac implantable electronic devices and cybersecurity. Expert Rev. Med. Devices.

[140] Crossley G.H., Boyle A., Vitense H., Chang Y., Mead R.H., Investigators C. (2011). The CONNECT (Clinical Evaluation of Remote Notification to Reduce Time to Clinical Decision) trial: The value of wireless remote monitoring with automatic clinician alerts. J. Am. Coll. Cardiol..

[141] Das S., Siroky G.P., Lee S., Mehta D., Suri R. (2021). Cybersecurity: The need for data and patient safety with cardiac implantable electronic devices. Heart Rhythm.

[142] Bour G., Lie A.W., Kok J.S., Markussen B., Moe M.E.G., Borgaonkar R. Security Analysis of the Internet of Medical Things (IoMT): Case Study of the Pacemaker Ecosystem. Proceedings of the International Joint Conference on Biomedical Engineering Systems and Technologies.

[143] Guo L., Dong M., Ota K., Li Q., Ye T., Wu J., Li J. (2017). A secure mechanism for big data collection in large scale internet of vehicle. IEEE Internet Things J..

[144] Muhammed T., Mehmood R., Albeshri A., Katib I. (2018). UbeHealth: A personalized ubiquitous cloud and edge-enabled networked healthcare system for smart cities. IEEE Access.

[145] Janbi N., Katib I., Albeshri A., Mehmood R. (2020). Distributed artificial intelligence-as-a-service (DAIaaS) for smarter IoE and 6G environments. Sensors.

[146] Vora J., Tanwar S., Tyagi S., Kumar N., Rodrigues J.J. FAAL: Fog Computing-Based Patient Monitoring System for Ambient Assisted Living. Proceedings of the 2017 IEEE 19th International Conference on e-Health Networking, Applications and Services (Healthcom).

[147] Malin B., Sweeney L. (2004). How (not) to protect genomic data privacy in a distributed network: Using trail re-identification to evaluate and design anonymity protection systems. J. Biomed. Inform..

[148] Beck E., Goin M.E., Ho A., Parks A., Rowe S. (2021). Critical digital literacy as method for teaching tactics of response to online surveillance and privacy erosion. Comput. Compos..

[149] Sarker I.H., Kayes A., Badsha S., Alqahtani H., Watters P., Ng A. (2020). Cybersecurity data science: An overview from machine learning perspective. J. Big Data.

[150] Kirk S. (2014). The Wearables Revolution: Is Standardization a Help or a Hindrance?: Mainstream technology or just a passing phase?. IEEE Consum. Electron. Mag..

[151] Xie M., Huang W., Yang L., Yang Y. (2016). VOAuth: A solution to protect OAuth against phishing. Comput. Ind..

[152] Navas J., Beltrán M. (2019). Understanding and mitigating OpenID Connect threats. Comput. Secur..

[153] Huda S., Islam M.R., Kottala V.N.V., Abawajy J.H. (2024). A Cyber Risk Assessment Approach to Federated Identity Management Framework Based Digital Healthcare System. Sensors.

[154] Altenstetter C. (2012). Medical device regulation in the European Union, Japan and the United States. Commonalities, differences and challenges. Innov. Eur. J. Soc. Sci. Res..

[155] Zieni B., Heckel R. TEM: A Transparency Engineering Methodology Enabling Users’ Trust Judgement. Proceedings of the 2021 IEEE 29th International Requirements Engineering Conference (RE).

[156] Zieni B., Spagnuelo D., Heckel R. Transparency by Default: GDPR Patterns for Agile Development. Proceedings of the International Conference on Electronic Government and the Information Systems Perspective.

[157] Siddiqui Z., Shukla V.K., Sharma N. (2024). A Review of the New European Medical Device Regulations. https://www.wjpr.net/abstract_show/25248.

[158] Melvin T., Torre M. (2019). New medical device regulations: The regulator’s view. Efort Open Rev..

[159] Carl A.K., Hochmann D. (2024). Impact of the new European medical device regulation: A two-year comparison. Biomed. Eng. Tech..

[160] Darrow J.J., Avorn J., Kesselheim A.S. (2021). FDA regulation and approval of medical devices: 1976–2020. JAMA.

[161] Beharry M. (2010). DBS: A UK (MHRA) regulatory perspective. Bioanalysis.

[162] Karthika B., Vijayakumar A. (2022). ISO 13485: Medical Devices—Quality Management Systems, Requirements for Regulatory Purposes. Medical Device Guidelines and Regulations Handbook.

[163] Tzanou M. (2023). Health Data Privacy Under the GDPR.

[164] Riad A.K.I., Barek M.A., Rahman M.M., Akter M.S., Islam T., Rahman M.A., Mia M.R., Shahriar H., Wu F., Ahamed S.I. Enhancing HIPAA Compliance in AI-driven mHealth Devices Security and Privacy. Proceedings of the 2024 IEEE 48th Annual Computers, Software, and Applications Conference (COMPSAC).

[165] Soman R., Onoufriou T., Votsis R., Chrysostomou C., Kyriakides M. Optimisation of Multi-type Sensor Placement for SHM Based on Application Demands. Proceedings of the IABSE Symposium Report.

[166] Soman R.N., Onoufrioua T., Kyriakidesb M.A., Votsisc R.A., Chrysostomou C.Z. (2014). Multi-type, multi-sensor placement optimization for structural health monitoring of long span bridges. Smart Struct. Syst..

[167] Sun X., Hou G., Wang Z. Sensitivity-Based Optimal Sensor Placement of Multi-Type Sensor. Proceedings of the Health Monitoring of Structural and Biological Systems.

[168] Chan P.Y., Ripin Z.M., Halim S.A., Arifin W.N., Yahya A.S., Eow G.B., Tan K., Hor J.Y., Wong C.K. (2022). Motion characteristics of subclinical tremors in Parkinson’s disease and normal subjects. Sci. Rep..

[169] Channa A., Ruggeri G., Ifrim R.C., Mammone N., Iera A., Popescu N. (2024). Cloud-Connected Bracelet for Continuous Monitoring of Parkinson’s Disease Patients: Integrating Advanced Wearable Technologies and Machine Learning. Electronics.

[170] Pratihar R., Sankar R. (2024). Advancements in Parkinson’s Disease Diagnosis: A Comprehensive Survey on Biomarker Integration and Machine Learning. Computers.

[171] Moore K., O’Shea E., Kenny L., Barton J., Tedesco S., Sica M., Crowe C., Alamäki A., Condell J., Nordström A. (2021). Older adults’ experiences with using wearable devices: Qualitative systematic review and meta-synthesis. JMIR MHealth UHealth.

[172] Rahme M., Folkeard P., Scollie S. (2021). Evaluating the accuracy of step tracking and fall detection in the Starkey Livio artificial intelligence hearing aids: A pilot study. Am. J. Audiol..

[173] Brognara L., Palumbo P., Grimm B., Palmerini L. (2019). Assessing gait in Parkinson’s disease using wearable motion sensors: A systematic review. Diseases.

[174] Barman J., Uswatte G., Ghaffari T., Sokal B., Byrom E., Trinh E., Brewer M., Varghese C., Sarkar N. (2012). Sensor-enabled RFID system for monitoring arm activity: Reliability and validity. IEEE Trans. Neural Syst. Rehabil. Eng..

[175] Barman J., Uswatte G., Sarkar N., Ghaffari T., Sokal B. Sensor-Enabled RFID System for Monitoring Arm Activity in Daily Life. Proceedings of the 2011 Annual International Conference of the IEEE Engineering in Medicine and Biology Society.

[176] Yared R., Negassi M.E., Yang L. Physical Activity Classification and Assessment Using Smartphone. Proceedings of the 2018 IEEE 9th Annual Information Technology, Electronics and Mobile Communication Conference (IEMCON).

[177] Song M., Kim J. (2017). An ambulatory gait monitoring system with activity classification and gait parameter calculation based on a single foot inertial sensor. IEEE Trans. Biomed. Eng..

[178] Cornacchia M., Ozcan K., Zheng Y., Velipasalar S. (2016). A survey on activity detection and classification using wearable sensors. IEEE Sens. J..

[179] Doherty A.R., Kelly P., Kerr J., Marshall S., Oliver M., Badland H., Hamilton A., Foster C. (2013). Using wearable cameras to categorise type and context of accelerometer-identified episodes of physical activity. Int. J. Behav. Nutr. Phys. Act..

[180] Liang B., Zheng L. A Survey on Human Action Recognition Using Depth Sensors. Proceedings of the 2015 International Conference on Digital Image Computing: Techniques and Applications (DICTA).

[181] Vahdani E., Tian Y. (2022). Deep learning-based action detection in untrimmed videos: A survey. IEEE Trans. Pattern Anal. Mach. Intell..

[182] Hadjidj A., Souil M., Bouabdallah A., Challal Y., Owen H. (2013). Wireless sensor networks for rehabilitation applications: Challenges and opportunities. J. Netw. Comput. Appl..

[183] Bijalwan V., Semwal V.B., Singh G., Mandal T.K. (2023). HDL-PSR: Modelling spatio-temporal features using hybrid deep learning approach for post-stroke rehabilitation. Neural Process. Lett..

[184] Nam Y.H., Bai Y., Lee J.A., Kim Y., Lee J.M., Meier N.F., Welk G.J. (2015). Validity Of Consumer-based Physical Activity Monitors In Semi-structured Activities: 982 Board# 3 May 27, 3: 15 PM-5: 15 PM. Med. Sci. Sport. Exerc..

[185] Case M.A., Burwick H.A., Volpp K.G., Patel M.S. (2015). Accuracy of smartphone applications and wearable devices for tracking physical activity data. JAMA.

[186] Kelly J.T., Campbell K.L., Gong E., Scuffham P. (2020). The Internet of Things: Impact and implications for health care delivery. J. Med. Internet Res..

[187] Tuckson R.V., Edmunds M., Hodgkins M.L. (2017). Telehealth. N. Engl. J. Med..

[188] Thakur N., Han C.Y. (2022). A simplistic and cost-effective design for real-world development of an ambient assisted living system for fall detection and indoor localization: Proof-of-concept. Information.

[189] Future M.R. (2023). Ambient Assisted Living Market Worth USD 23.5 Billion at a CAGR of 19.1% by 2030. https://www.globenewswire.com/news-release/2023/03/02/2619001/0/en/Ambient-Assisted-Living-Market-Worth-USD-23-5-Billion-at-a-CAGR-of-19-1-by-2030-Report-by-Market-Research-Future-MRFR.html.

[190] Cryer L., Shannon S.B., Van Amsterdam M., Leff B. (2012). Costs for ‘hospital at home’patients were 19 percent lower, with equal or better outcomes compared to similar inpatients. Health Aff..

[191] Klein S. (2022). “Hospital at Home” Programs Improve Outcomes, Lower Costs But Face Resistance from Providers and Payers.

[192] Megido I., Sela Y., Grinberg K. (2023). Cost effectiveness of home care versus hospital care: A retrospective analysis. Cost Eff. Resour. Alloc..

[193] The National Council on Aging Falls Prevention Facts. https://www.ncoa.org/news/resources-for-reporters/get-the-facts/falls-prevention-facts/.

[194] Centers for Disease Control and Prevention Older Adult Falls Reported by State. https://www.cdc.gov/falls/data-research/facts-stats/index.html.

[195] Hassan C.A.U., Karim F.K., Abbas A., Iqbal J., Elmannai H., Hussain S., Ullah S.S., Khan M.S. (2023). A Cost-Effective Fall-Detection Framework for the Elderly Using Sensor-Based Technologies. Sustainability.

[196] Living M.S. (2023). The Financial Impact of Fall Detection Wearable Devices on Senior Living Communities. https://www.mcknightsseniorliving.com/home/columns/marketplace-columns/the-financial-impact-of-falls-detection-wearable-devices-on-senior-living-communities-noi/.

[197] Giovanelli D., Milosevic B., Brunelli D., Farella E. Enhancing Bluetooth Low Energy with wake-up radios for IoT applications. Proceedings of the 2017 13th International Wireless Communications and Mobile Computing Conference (IWCMC).

[198] Corbellini G., Schmid S., Mangold S. Two-Way Communication Protocol Using Bluetooth Low Energy Advertisement Frames. Proceedings of the 1st International Workshop on Experiences with the Design and Implementation of Smart Objects.

[199] Akrivopoulos O., Chatzigiannakis I., Tselios C., Antoniou A. On the Deployment of Healthcare Applications over Fog Computing Infrastructure. Proceedings of the 2017 IEEE 41st annual computer software and applications conference (COMPSAC).

[200] Qaim W.B., Ometov A., Molinaro A., Lener I., Campolo C., Lohan E.S., Nurmi J. (2020). Towards energy efficiency in the internet of wearable things: A systematic review. IEEE Access.

[201] Zhou D., Li Z., Zhu J., Zhang H., Hou L. (2020). State of health monitoring and remaining useful life prediction of lithium-ion batteries based on temporal convolutional network. IEEE Access.

[202] Kalantarian H., Sideris C., Mortazavi B., Alshurafa N., Sarrafzadeh M. (2016). Dynamic computation offloading for low-power wearable health monitoring systems. IEEE Trans. Biomed. Eng..

[203] Mansourkiaie F., Ismail L.S., Elfouly T.M., Ahmed M.H. (2017). Maximizing lifetime in wireless sensor network for structural health monitoring with and without energy harvesting. IEEE Access.

[204] Nasr M., Islam M.M., Shehata S., Karray F., Quintana Y. (2021). Smart healthcare in the age of AI: Recent advances, challenges, and future prospects. IEEE Access.

[205] Nižetić S., Šolić P., Gonzalez-De D.L.d.I., Patrono L. (2020). Internet of Things (IoT): Opportunities, issues and challenges towards a smart and sustainable future. J. Clean. Prod..

[206] Wang C., He T., Zhou H., Zhang Z., Lee C. (2023). Artificial intelligence enhanced sensors-enabling technologies to next-generation healthcare and biomedical platform. Bioelectron. Med..

[207] Foy K. (2023). New Tools Are Available to Help Reduce the Energy That AI Models Devour. https://news.mit.edu/2023/new-tools-available-reduce-energy-that-ai-models-devour-1005.

[208] Chuah S.H.W., Rauschnabel P.A., Krey N., Nguyen B., Ramayah T., Lade S. (2016). Wearable technologies: The role of usefulness and visibility in smartwatch adoption. Comput. Hum. Behav..

[209] Park E. (2020). User acceptance of smart wearable devices: An expectation-confirmation model approach. Telemat. Inform..

[210] Sui A., Sui W., Liu S., Rhodes R. (2023). Ethical considerations for the use of consumer wearables in health research. Digit. Health.

[211] Ledger D., McCaffrey D. (2014). Inside wearables: How the science of human behavior change offers the secret to long-term engagement. Endeav. Partners.

[212] Piwek L., Ellis D.A., Andrews S., Joinson A. (2016). The rise of consumer health wearables: Promises and barriers. PLoS Med..

